# Tumor Necrosis Factor Receptor-1 Associated Periodic Syndrome: Case Report and Review of an Auto-inflammatory Disorder

**DOI:** 10.7759/cureus.3916

**Published:** 2019-01-19

**Authors:** Javier Quintero, Jihan Saba, Carlos Garcia

**Affiliations:** 1 Internal Medicine, University of Miami Miller School of Medicine Regional Campus, Fort Lauderdale, USA; 2 Internal Medicine, University of Miami at Holy Cross Hospital, Fort Lauderdale, USA

**Keywords:** tumor necrosis factor receptor-1 associated periodic syndrome, traps, auto-inflammatory disorder, interleukin 1 antagonist, cyclical fever syndrome

## Abstract

Tumor necrosis factor receptor-1 associated periodic syndrome (TRAPS) is a very rare, hereditary, auto-inflammatory disorder caused by a genetic mutation within the tumor necrosis factor receptor superfamily member one-A (TNFRSF1A) gene, resulting in unregulated, systemic inflammation. We will present a patient who suffered through years of multiple medical problems of unknown etiology and will describe the process leading to the diagnosis of TRAPS. It is important to consider this syndrome as a rare but probable diagnosis in patients who lack a unifying explanation for multiple inflammatory symptoms.

## Introduction

Tumor necrosis factor receptor-1 associated periodic syndrome (TRAPS) is a type of auto-inflammatory disorder and periodic fever syndrome with a prevalence of one per million. TRAPS is inherited in autosomal dominant fashion with incomplete penetrance [1-3]. In all types of auto-inflammatory diseases, the main pathologic process resides within the patient’s own innate immunity, resulting in aberrant over-expression of the immune system. This is different from acquired immunity in autoimmune disorders. The diagnoses for most of these auto-inflammatory syndromes are confirmed by gene analysis. The genetic culprit behind TRAPS lies within a mutation that encodes for the tumor necrosis factor (TNF) receptor superfamily member one-A (TNFRSF1A) gene [3-4].

## Case presentation

A 48-year-old Hispanic female with no significant medical history presented to the clinic with a two-year history of multiple medical complaints, including occasional low-grade fevers, intermittent chills, night sweats, recurrent episodes of left eye pain with redness, pleuritic chest pains, intermittent abdominal pain, diffuse myalgias and achiness on the left side of her face, fatigue, hair loss, and unintentional weight loss of 30 pounds. A review of systems revealed insomnia and a pruritic rash on her right hand and right foot that began two days prior to presentation.

The patient denied recent travel, oral/nasal ulcers, joint swelling, morning stiffness, Raynaud’s, photosensitivity, malar rash, or sicca symptoms. A recent short course of oral glucocorticoids helped with her pleuritic pain. She had a history of eight miscarriages and mentioned that past workup had been negative for antiphospholipid syndrome. Multiple specialists had evaluated her over the past two years without a clear unifying diagnosis. The patient denied a family history of malignancy, connective tissue disease, or any autoimmune disorder. 

Her physical exam revealed a temperature of 98.8°F, blood pressure of 136/94, and heart rate of 110. The recent range in temperatures from outpatient encounters was within the range of 98.8-99.9°F. The patient was in no acute distress and appeared well-nourished. No oral or nasal lesions were appreciated, and her oropharynx was clear. Her left eye appeared injected and her neck was without adenopathy or thyromegaly. The cardiopulmonary exam was unremarkable. Diffuse tenderness was noted on the left metacarpophalangeal joints, wrist, elbow, and shoulder but the range of motion was normal and no deformities or joint swelling was noted. The skin exam revealed a dry patch of 5-centimeter diameter on the dorsum of the right foot without swelling. No Raynaud’s, telangiectasias, or skin ulcers were noted.

Recent cardiac testing revealed a negative stress test and echocardiogram, showing a small pericardial effusion with normal ventricular function. Recent labs were remarkable for an elevated erythrocyte sedimentation rate (ESR) of 106 mm/h, an elevated C-reactive protein (CRP) of 112 mg/L and a rheumatoid factor of 24 IU/ml. Anti-cyclic citrullinated peptide and antinuclear antibody panel, including antibodies for double-stranded deoxyribonucleic acid (DNA), Scl-70, Smith, RNP, SS-A, and SS-B were negative. Levels of complement C3 and C4, ferritin, creatine kinase, and serum protein electrophoresis were noted within normal range. Erythrocyte sedimentation rate (ESR) and CRP levels normalized with a course of oral steroids but subsequently increased after cessation of therapy.

The patient was referred to ophthalmology and diagnosed with left eye scleritis that resolved with steroid eye drops. The patient was evaluated by oncology and infectious disease and thought not to have an underlying malignancy or infection. Considering the constellation of symptoms, the patient was then evaluated for a periodic fever syndrome. Genetic testing ultimately revealed and confirmed the diagnosis of TRAPS. The patient was then started on treatment with an interleukin-one (IL-one) antagonist canakinumab, resulting in significant improvement of myalgias, achiness, fatigue, chills, and hair loss. Within eight weeks of initiating therapy, she reported great relief of her symptomatic burden with the resolution of chest pain, rashes, scleritis, and fevers. Her ESR and CRP both normalized. Notably, the patient's 26-year-old nephew had experienced similar symptoms since childhood, including migratory myalgias, cyclical chills, and night sweats. The identification and successful treatment of our patient's disease led to genetic testing, confirmation and successful treatment of her nephew with the IL-one inhibitor canakinumab.

## Discussion

Although it was originally found that most TNFRSF1A mutations related to TRAPS occurred in patients of Irish or Scottish ancestry; this syndrome has been described in many other diverse populations. This suggests that the diagnosis should not be excluded based on a patient’s ancestry [1-2]. Both our patient and her nephew are Hispanic, from South America, and had no first degree relatives that suffered from TRAPS or any other inflammatory or autoimmune disease. Family history is usually positive in TRAPS; although some carriers may be entirely asymptomatic [4]. The median age at the time of diagnosis is three years old and most patients will present in their first decade but 10% present over the age of 30 years. Interestingly, a marked disparity in age of onset may occur within the same family [2,5].

The common signs and symptoms of TRAPS include recurrent fever, conjunctivitis, arthritis, chest pain, pericarditis, myalgias, and an erythematous skin rash. A characteristic feature of the myalgias is its pattern of centrifugal spread throughout the body. Arthralgia of the large joints is more common than synovitis, which is asymmetric and non-erosive. In the absence of underlying infection, malignancy, or other autoimmune disorders, the most common clinical manifestation of TRAPS is a recurrent fever. The timing of fever is variable, as it can last up to five days or continue for weeks at a time [1,6]. Glucocorticoids help limit acute flares but only serve as a temporary measure. No head-to-head trials currently exist, but data tends to favor IL-one antagonists as the first-line therapy over anti-TNF agents [7-9].

## Conclusions

The diagnosis of TRAPS should be considered in patients with a recurrent fever of unknown origin, along with any of the clinical features related to chronic inflammation described earlier, after other, more common causes, such as infections, malignancy, and even auto-immune disorders have been eliminated as diagnostic possibilities. There are no specific laboratory tests but inflammatory markers, such as CRP and ESR, will be elevated during an acute disease flare. Specific testing for the TNFRSF1A gene mutation confirms the diagnosis of TRAPS. The main goal of treatment consists of controlling symptoms, decreasing systemic inflammation, and preventing long-term complications, such as the progression of cardiovascular disease and even systemic amyloidosis. The positive outcomes with IL-one antagonist therapy experienced by both our patient and her nephew help support the use of similar therapy in others suffering from this rare and crippling auto-inflammatory disorder.
